# Effect of enzymatic chemo-mechanical agents on adhesion of composite resin to dentin of permanent teeth: an in vitro study

**DOI:** 10.1007/s40368-024-00949-9

**Published:** 2024-11-01

**Authors:** A. S. Coelho, L. Vilhena, I. Amaro, A. Melo, A. Paula, C. M. Marto, M. M. Ferreira, A. Ramalho, E. Carrilho

**Affiliations:** 1 Institute of Integrated Clinical Practice, Faculty of Medicine, University of Coimbra, Coimbra, Portugal; 2 Institute for Clinical and Biomedical Research (iCBR), Area of Environment, Genetics and Oncobiology (CIMAGO), Faculty of Medicine, University of Coimbra, Coimbra, Portugal; 3 CNC.IBILI Consortium, Faculty of Medicine, University of Coimbra, Coimbra, Portugal; 4 Centre for Innovative Biomedicine and Biotechnology (CIBB), University of Coimbra, Coimbra, Portugal; 5 Centre for Mechanical Engineering, Materials and Processes (CEMMPRE), University Coimbra, 3004‐516 Coimbra, Portugal; 6 Institute of Experimental Pathology, Faculty of Medicine, University of Coimbra, Coimbra, Portugal; 7 Institute of Endodontics, Faculty of Medicine, University of Coimbra, Coimbra, Portugal

**Keywords:** Dental bonding, Brix 3000™, Dental caries, Papacárie Duo^®^, Shear bond strength

## Abstract

**Purpose:**

To evaluate and compare the effect of two enzymatic chemo-mechanical caries removal agents with conventional caries removal using rotatory instruments on the adhesion of composite resin to dentin of permanent teeth.

**Methods:**

The sample comprised 30 permanent molars with caries lesions extending to the dentin, randomly distributed into three groups (*n* = 10 each): 1—Caries removal with rotary instruments (control group); 2—Caries removal with Papacárie Duo^®^ (F&A Laboratório Farmacêutico, São Paulo, Brazil); 3—Caries removal with Brix 3000™ (Brix S.R.L., Carcarañá, Argentina). After caries removal, the specimens were rinsed and dried. Scotchbond Universal™ adhesive (3 M, Saint Paul, Minnesota, USA) was actively applied in self-etch mode and light-cured. Resin composite increments were applied using a silicone mold (3 × 3 × 2 mm) and light-cured. Shear bond strength (MPa), work-to-debonding (J/m^2^), and shear modulus (kPa) were evaluated. For statistical analysis, the level of significance was set at 5%.

**Results:**

The control group presented significantly higher shear bond strength values (8.50 ± 2.69 MPa) compared to the Brix 3000™ group (5.72 ± 1.55 MPa, *p* = 0.008). There were no significant differences between Papacárie Duo^®^ (6.66 ± 0.86 MPa) and the other groups (*p* > 0.05). Regarding work-to-debonding, the Papacárie Duo^®^ group had a significantly higher result (2944.41 ± 450.21 J/m^2^) than the Brix 3000™ group (1189.41 ± 504.13 J/m^2^, *p* < 0.001) and the control group (967.10 ± 270.01 J/m^2^, *p* < 0.001). Concerning shear modulus, the control group showed a significantly higher result (558.67 ± 168.96 kPa) than the Brix 3000™ group (339.79 ± 143.78 kPa, *p* = 0.008) and the Papacárie Duo^®^ group (223.04 ± 127.30 kPa, *p* < 0.001).

**Conclusion:**

While the application of Papacárie Duo^®^ did not negatively affect composite resin adhesion to dentin of permanent teeth, the application of Brix 3000™ reduced adhesive forces, potentially limiting its clinical use. Further investigations are warranted to elucidate the effects of these materials on dentin substrate, particularly through clinical studies.

## Introduction

Dental caries is one of the most prevalent oral cavity diseases worldwide. It is an infectious, chronic, post-eruptive, and multifactorial disease dependent on several factors, including diet, fluoride exposure, host susceptibility, oral microflora, oral hygiene, social and cultural factors, as well as quality and quantity of saliva (American Academy of Pediatric Dentistry 2014; Cheng et al. 2022; Mathur and Dhillon 2018; Pitts et al. 2017).

The treatment of carious lesions aims to eliminate or control pathogenic factors, remove infected carious tissue, and restore tooth structure, ultimately providing a functional and aesthetic solution. The most common method for treating these lesions involves removing the carious tissue using rotary instruments, followed by restorative treatment. This approach allows for effective biofilm control and protection of the pulpo-dentinal complex. However, this process may face challenges due to systemic factors, lesion extension, the type of tissues affected (enamel and/or dentin), and technical sensitivity (Cheng et al. 2022; Pitts et al. 2017; Ramos-Gomez et al. 2010).

In recent years, scientific research has explored the development of alternative methods for caries removal, including enzymatic chemo-mechanical removal techniques. These methods involve the use of products classified into two groups: (1) sodium hypochlorite associated with amino acids; (2) proteolytic enzymes-based products. The chemical reaction of these agents softens the carious dentin, making it easier to remove with manual instruments (Cardoso et al. 2020; Maashi et al. 2023; Souza et al. 2022).

Recent studies have primarily focused on the development and examination of enzymatic products. This emphasis stems from the instability and aggressiveness of sodium hypochlorite-based products towards healthy tissues, which also necessitate longer treatment times and incur higher costs (Cardoso et al. 2020; Souza et al. 2022).

In comparison to the conventional method of caries removal with rotary instruments, the use of chemo-mechanical agents offers several advantages. These include tissue selectivity, as both healthy and affected tissues are preserved due to the presence of antiprotease alpha-1-antitrypsin, inhibiting proteolysis. Additionally, these agents prevent the complete obliteration of dentinal tubules by the smear layer and reduce instances where local anesthesia is required (Alkhouli et al. 2020; Sajjad et al. 2022; Santos et al. 2020).

While conventional methods employing rotary instruments remain the most widely utilized due to their speed and effectiveness in removing carious tissue, the associated factors of vibration, heat, and noise often contribute to patient discomfort and anxiety. Chemo-mechanical methods for caries removal, on the other hand, have been associated with a reduction in anxiety, as well as less pain and discomfort for the patient. Consequently, these methods may prove particularly beneficial for children and patients with a high degree of anxiety and/or phobias. However, it is essential to acknowledge that the use of chemo-mechanical methods requires a longer treatment time, a factor that should be taken into consideration (Cardoso et al. 2020; Maashi et al. 2023; Sharma et al. 2023; Zou et al. 2022).

Among the enzymatic products, Papacárie Duo^®^ (F&A Laboratório Farmacêutico, São Paulo, Brazil) and Brix 3000™ (Brix S.R.L., Carcarañá, Argentina) stand out. Papacárie^®^ was developed in 2003, becoming the first enzyme-based product introduced in the market. It contains 10% papain (6000 IU/mg), a proteolytic enzyme capable of degrading collagen fibers, with anti-inflammatory, antibacterial, and bactericidal properties. Additionally, it includes chloramine, providing antiseptic action and contributing to the removal of denatured tissues (Maashi et al. 2023; Souza et al. 2022). In 2011, a new version of the product was introduced (Papacárie Duo^®^), featuring higher viscosity for improved handling, eliminating the need for refrigeration, and extending durability (Adham et al. 2021).

Brix 3000™ was developed in 2012, containing 10% papain (30,000 IU/mg) and a mechanism of action named Encapsulated Buffer Emulsion (EBE). This mechanism provides greater stability to the product, fixing the gel, and maintaining an ideal pH to immobilize the enzymes. It enhances the effectiveness of the proteolytic activity, promoting the breakdown of collagen fibers in decayed tissue, and reducing the dissolution of the product upon contact with saliva and blood (Alkhouli et al. 2020; Maashi et al 2023; Ismail and Al Haidar 2019).

Nevertheless, the success and longevity of restoration depend not only on the method used for caries removal but also on the remaining dental substrate, the type of tooth isolation (relative or absolute), the adhesive system applied, and the restorative material used. Properly sealing the tooth structure is essential to prevent secondary caries, marginal infiltration, and/or pulpal irritation (Cardoso et al. 2020; Sajjad et al. 2022).

The effect of applying enzymatic chemo-mechanical caries removal methods on the adhesion of composite resin to dentin is not fully understood. Due to its complex heterogeneous composition, adhesion to dentin is more unpredictable than to enamel. Therefore, it is essential to investigate the effect of applying these products on dentin adhesion (Donmez et al. 2022; Sajjad et al. 2022).

Thus, the aim of this in vitro study was to evaluate and compare the effects of two enzymatic chemo-mechanical caries removal agents (Papacárie Duo^®^ and Brix 3000™) compared to conventional caries removal with rotatory instruments on the adhesion of composite resin to the dentin of permanent teeth. Three different adhesion parameters were evaluated: shear bond strength (primary outcome, N/m^2^ or Pa) and work-to-debonding (J/m^2^) and shear modulus (KPa) (secondary outcomes). The established null hypothesis was that there are no differences between the control group (conventional caries removal with rotary instruments) and the test groups (chemo-mechanical caries removal), for any of the tested parameters.

## Materials and methods

This study received approval from the Ethics Committee of the Faculty of Medicine of the University of Coimbra (CE-161/2022), adhering to applicable laws, regulations, and the 1964 Declaration of Helsinki and its later amendments. All participants read, understood, and signed an informed consent form.

The study involved 30 permanent molars with cavitated class I or class II carious lesions, displaying dentin involvement, and extracted due to mobility, fracture, and/or impossibility of restoration. The cavity needed to extend at least halfway from the enamel-dentin junction to the pulp chamber and, after caries removal, to meet the inclusion criteria and allow for the planned tests to be conducted.

Periapical radiographs were taken prior to tooth extraction, which allowed for the evaluation of the depth of the lesion and its proximity to the pulp chamber. During the procedure, the extent of the cavity was visually assessed and verified through probing to ensure it extended at least halfway from the enamel-dentin junction to the pulp chamber. If, after caries removal, there was any uncertainty regarding the extension into the dentin, an additional radiograph was taken to confirm the appropriate depth.

Exclusions comprised teeth with carious lesions involving the pulp, those with root caries lesions, and those with caries lesions affecting only the enamel.

After extraction, teeth were immediately rinsed with running water to remove all blood and soft tissues. They were then stored in distilled water for a maximum period of six months, following ISO/TS 11405:2015 (Dental materials—testing of adhesion to tooth structure) guidelines (International Organization for Standardization 2015), until subjected to the tests.

All specimens were submerged in autopolymerizing acrylic resin (Probase, Ivoclar Vivadent, Schaan, Liechtenstein), within a 5 × 2.5 cm polyvinyl chloride (PVC) cylinder. Only the crown portion was not submerged.

The permanent teeth were randomly distributed into three groups (*n* = 10 each):

Group 1 (control group): Conventional mechanical caries removal with rotary instruments;

Group 2: Chemo-mechanical caries removal with Brix 3000^TM^;

Group 3: Chemo-mechanical caries removal with Papacárie Duo^®^.

To standardize the cavity configuration, before proceeding with caries removal, the decayed enamel layer and adjacent walls were eliminated using a water-cooled diamond bur (FG 801–014, Diaswiss^®^, Nyon, Switzerland). All caries removal procedures with the three chosen methods, as well as adhesive procedures, were performed on the pulpal wall.

Caries from group 1 (control group) were removed using a water-cooled spherical diamond bur for turbine (FG 801–014, Diaswiss^®^), until a hard consistency of the dentin was achieved by probing.

For group 2, Brix 3000™ was applied to remove caries. Following the manufacturer's recommendations, the product was placed in the decayed cavity for 2 min. Using an excavator, carious tissue was then removed with pendular movements and without pressure. This process was repeated until all infected tissue was completely removed, typically requiring between one and three applications. Finally, the specimens were rinsed with water for 20 s and then dried with an air jet.

For group 3, Papacárie Duo^®^ was applied to remove the caries. Following the manufacturer's recommendations, the product was placed in the decayed cavity for 30 s. Subsequently, using an excavator (EXC125/6, HuFriedy Group, Chicago, Illinois, USA), carious tissue was removed with pendular movements and without pressure. This process was repeated until all infected tissue was completely removed, typically requiring between one and three applications. Finally, the specimens were rinsed with water for 20 s and then dried with an air jet.

One experienced operator performed caries removal, assessing the complete elimination of the infected dentin based on tactile criteria (tissue hardness and roughness) and visual criteria (tissue pigmentation), while two additional experienced researchers observed and ensured concurrence with the operator's decisions and observations throughout the procedures.

After the removal of carious lesions, all teeth underwent the same restoration protocol. The Scotchbond Universal™ adhesive (3 M, Saint Paul, Minnesota, USA) was applied actively with a microbrush for 20 s, followed by air blasting for 10 s and light curing for 40 s using the SmartLite Focus unit (1000 mW/cm^2^ ± 10%, Dentsply Sirona, Charlotte, North Carolina, USA). The light intensity was verified with a radiometer (Bluephase^®^ meter, Ivoclar Vivadent). Using a 3 × 3 × 2 mm silicone mold on the sealed dentin, 1–2 increments of composite resin (Admira^®^ Fusion, VOCO, Cuxhaven, Germany) were applied and light-cured for 40 s.

The composition and manufacturer details of the materials used in this study are presented in Table 1.

**Table 1:** Enzymatic chemo-mechanical caries removal products used in the study

Products	Composition	Lot	Manufacturer
Brix 3000™	Papain (30,000 UI/mg); polyethylene glycol; citrus pectin; triethanolamine; Sorbitan monooleate; DISODIUM phosphate; monopotassium phosphate; Toluidine blue; distilled water	L49	Brix S.R.L., Carcarañá, Argentina
Papacárie Duo^®^	Papain (6000 UI/mg); chloramine T; Blue coloring; polyethylene glycol 400; polyethylene glycol 4000; Propylene glycol	9574	F&A Laboratório Farmacêutico, São Paulo, Brazil
Scotchbond Universal™	10-MDP, dimethacrylate, HEMA, methacrylate modified polyalkenoic acid copolymer, filler, ethanol, water, silane, initiators (pH 2.7)	7,910,512	3 M, Saint Paul, Minnesota, USA
Admira^®^ Fusion	Silicon dioxide (fillers and resin matrix)	2,139,695	VOCO, Cuxhaven, Germany

**Fig. 1:**
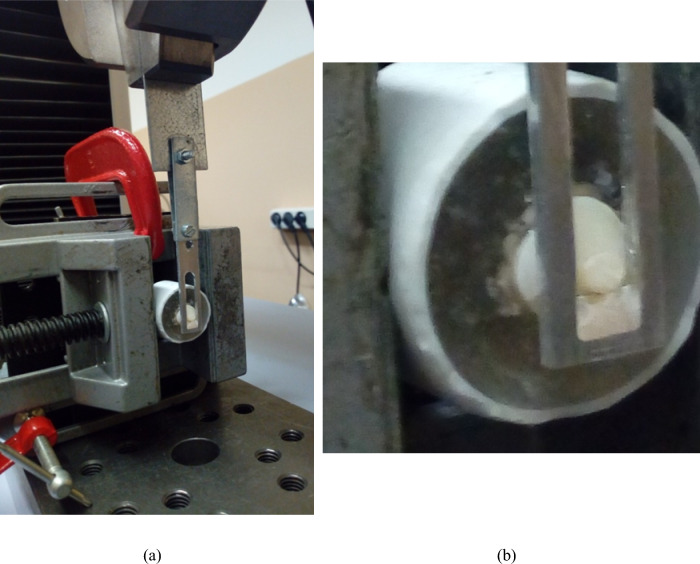
Specimen positioning on the Shimadzu universal testing machine: **a** general view; **b** joint detail

To assess the parameters providing information about the joint performance of the dental adhesive system, including shear bond strength (MPa), work-to-debonding (J/m^2^) and shear modulus (kPa) in accordance with ISO 29022:2013 (International Organization for Standardization 2013), a universal electromechanical testing machine (Shimadzu Autograph AG-X-1kN, Shimadzu Corporation, Kyoto, Japan) with a 5kN load cell was employed. The specimens were securely positioned in the equipment, with a sandpaper sheet wrapped around them to enhance friction between the sample and the support, preventing movement. A small guillotine-shaped blade was positioned on the upper brace and moved parallel to the adhesive joint between the dentin and the composite resin until the detachment of the restorative material occurred (Fig. 1). The travel speed was set at 0.5 mm/min.

**Fig. 2:**
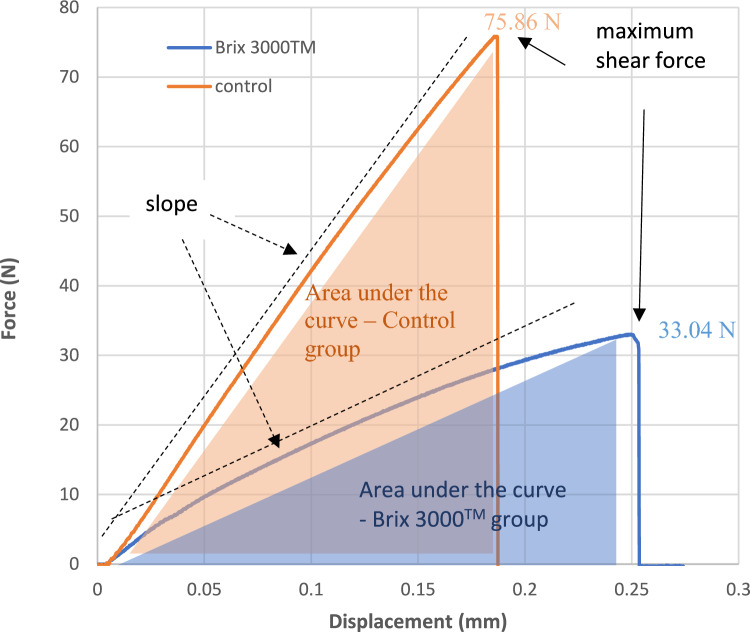
Example of force vs displacement curves for two tests performed using Brix 3000™ and control groups, showing the maximum shear force, slope and area under the curves force vs. displacement

As an example, and to elucidate the calculation of adhesion parameters, Fig. 2 shows force vs. displacement curves for two different tests performed using Brix 3000™ and the control group. As is visible from the behaviour of the two curves, an increase in displacement of the adhesive joint can be observed as the force increases. If, for any reason, the load is removed, in the area where the curve is linear, the deformation would disappear, as we are in the elastic region. It can be observed that the maximum force before rupture for Brix 3000TM is around 33.04 N, while for the Control group it is 75.86 N. This value, after being normalized by the area of the glued joint (3 mm diameter), indicates the value of the shear bond strength (4.67 MPa and 10.73 MPa, respectively). The slope of the curve provides another important parameter, which is the stiffness or shear modulus of that connection. Upon examination of Fig. 2 and for the examples analysed, it can be concluded that the shear modulus of the control test (631.13 kPa rad^−1^) exhibits higher rigidity than that of the Brix 3000™ (252.68 kPa rad^−1^). The area under each curve represents the energy absorbed until rupture occurs and indicates the durability of the adhesive joint subjected to repeated stresses. For the cases analysed, the work-to-debonding for the control group is slightly higher at 1001.65 J/m^2^, while for the Brix 3000™ it is 701.75 J/m^2^.

The assessment of failure mode (cohesive, adhesive, and mixed failures) was conducted through microscopic observation, employing a 3D Hirox digital microscope (Hirox, Tokyo, Japan) and a Hitachi SU3800 Scanning Electron Microscope (Hitachi, Tokyo, Japan). Energy-Dispersive X-ray Spectroscopy (EDS) with an XFlash Detector 610 M (Bruker, Billerica, Massachusetts, USA) was also used to identify all the chemical elements present at the joint surface. When the EDS module was used, the voltage was increased to 15 kV, and the spot size to 80. Before carrying out the SEM/EDS analysis, the specimens were dehydrated in an oven at 50 °C for 12 h. They were then placed and mounted in a sample holder where carbon tape bridges were used from the conductive base of the sample holder to the dentin surface. Subsequently, a 108 Auto Sputter Coater V2, 115VAC (Cressington, Watford, United Kingdom) was used, applying a gold coating with a thickness between approximately 3–5 nm (deposition rate 0.33 A°/s) to the dentine to create a conductive surface. The 3D digital microscope was solely used as a primary means of observation and to identify the area of interest to be observed using Scanning Electron Microscopy. Due to the costs associated with performing the gold sputter coating and SEM/EDS analysis, only two specimens from each group were analyzed by SEM/EDS.

Adhesive failure is defined as when the rupture occurs exclusively at the interface between dentin and the composite resin. Cohesive failure is identified when rupture occurs within the dentin or inside the composite resin cylinder. Mixed failure is recognized when components of both types of failure are detectable.

For data analysis, IBM^®^ SPSS^®^ v.29.0 (IBM Corporation, Armonk, New York, USA) statistical software was used. Normality was assessed using the Shapiro–Wilk test, and homogeneity of variances was tested using Levene's test. All data were analyzed using one-way ANOVA with a post-hoc Tukey test. The level of significance was set at 5%.

## Results

Statistically significant differences were found between groups regarding shear bond strength (*p* = 0.010), work-to-debonding (*p* < 0.001), and shear modulus (*p* < 0.001).

**Fig. 3:**
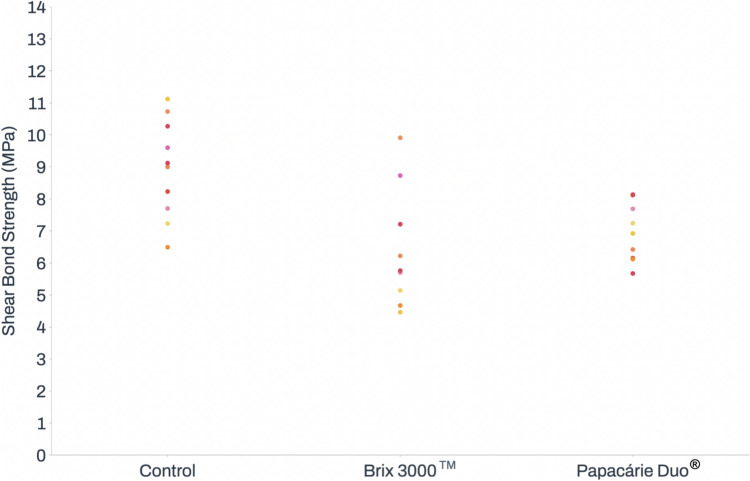
Effect of Brix 3000™ and Papacárie Duo^®^ on shear bond strength

The samples from the control group exhibited significantly higher shear bond strength (8.50 ± 2.69 MPa) than the Brix 3000™ group (5.72 ± 1.55 MPa, *p* = 0.008). No differences were found in relation to the Papacárie Duo^®^ group, whose samples showed an average shear bond strength of 6.66 ± 0.86 MPa. The results regarding shear bond strength are presented in Fig. 3.

**Fig. 4:**
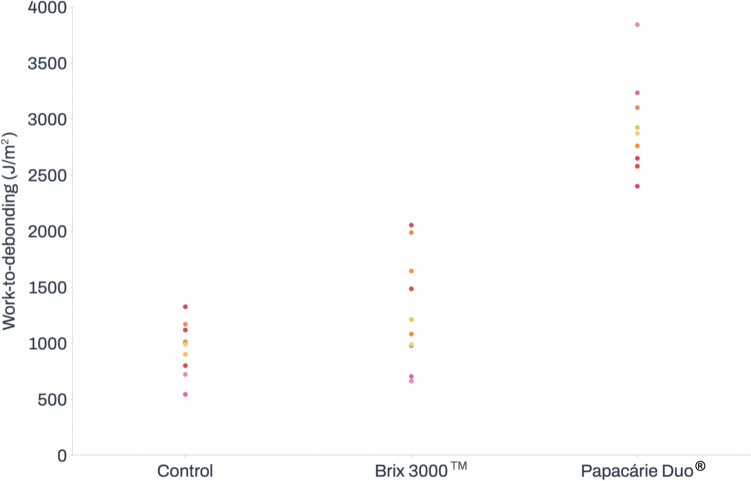
Effect of Brix 3000™ and Papacárie Duo^®^ on work-to-debonding

The Papacárie Duo^®^ group showed the highest work-to-debonding results. Statistically significant differences were found between the Papacárie Duo^®^ group (2944.41 ± 450.21 J/m^2^) and the control group (967.10 ± 270.01 J/m^2^, *p* < 0.001), as well as between the Papacárie Duo^®^ group and the Brix 3000™ group (1189.41 ± 504.13 J/m^2^, *p* < 0.001). Work-to-debonding results are presented in Fig. 4.

**Fig. 5:**
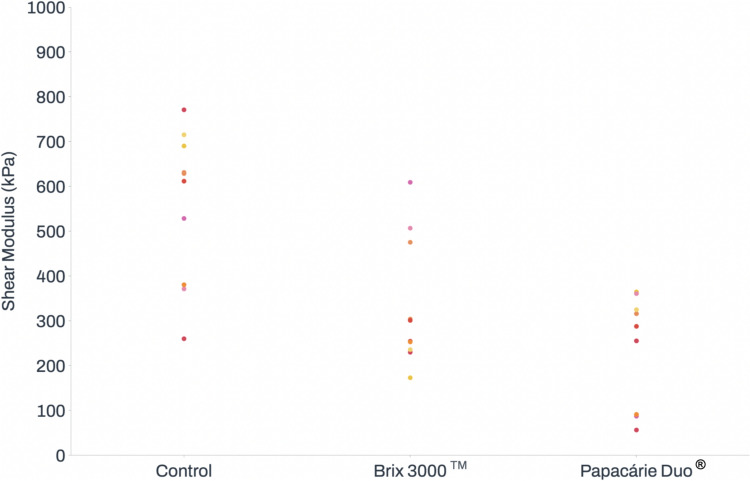
Effect of Brix 3000™ and Papacárie Duo^®^ on shear modulus

In terms of the shear modulus, statistically significant differences were identified between the control group (558.67 ± 168.96 kPa) and the test groups: Brix 3000™ (339.79 ± 143.78 kPa, *p* = 0.008) and Papacárie Duo^®^ (223.04 ± 127.30 kPa, *p* < 0.001). No significant differences were found between the test groups. The results regarding shear modulus are presented in Fig. 5.

**Fig. 6:**
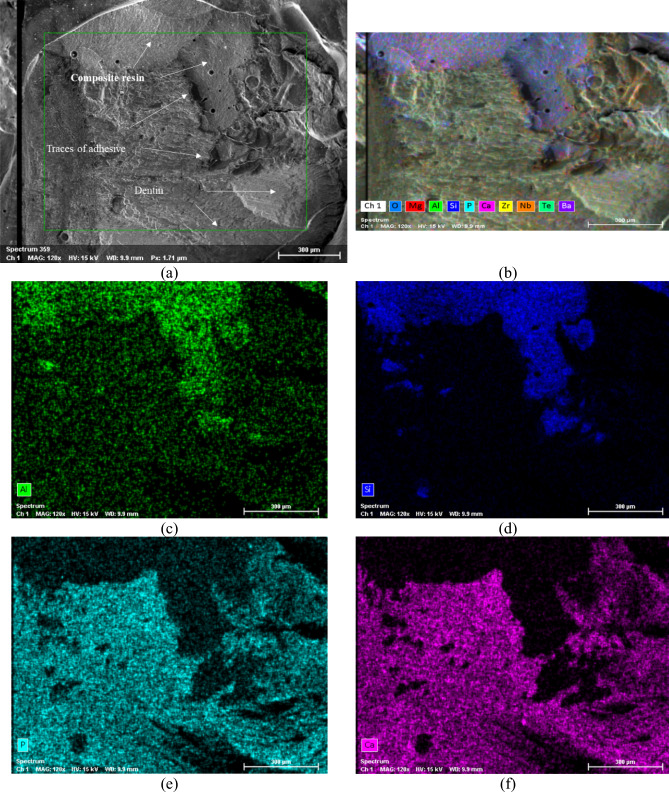
SEM/EDS mapping of the elements’ distribution at the adhesive joint for the Brix 3000™ group after shear bond strength test (specimen A3): **a** SEM micrograph showing the adhesive; **b** overall distribution of all chemical elements; **c** Al distribution; **d** Si distribution; **e** P distribution; **f** Ca distribution

Figure 6a shows a scanning electron microscope (SEM) micrograph of the bonded joint after performing the shear bond strength (SBS) test for the Brix 3000™ group (specimen A3). A closer analysis of the surface of the joint reveals different areas where remnants of the composite resin are likely to be identified, along with traces of adhesive visible at the interface, and parts of the dentin structure, indicating a mixed failure.

Figure 6b–f display the Energy-Dispersive X-ray Spectroscopy (EDS) mapping for the SEM micrograph depicted in Fig. 6a, enabling the identification of all chemical elements present at the surface.

**Fig. 7:**
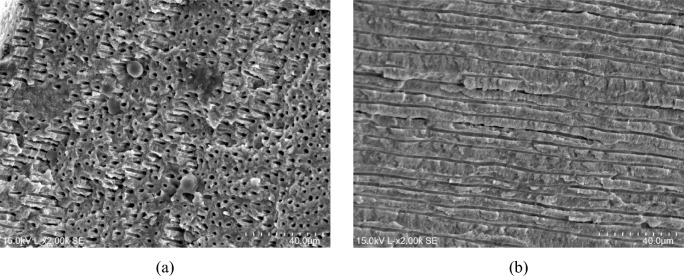
SEM micrographs showing part of the dentinal structure at the adhesive joint region after the shear bond strength test, where it is possible to observe the dentinal tubules: **a** transverse fracture (×2.00 k); **b** longitudinal fracture (×2.00 k)

Upon observing the EDS mapping, it is evident to distinguish the different regions mentioned above. Figure 6c and d correspond to the areas where remnants of the composite resin remain attached to the bonded joint. This is apparent from the predominance of Al and Si chemical elements, which are constituents of the composite resin. As for the regions depicted in Fig. 6e and f, they show a predominance of chemical elements P and Ca, respectively, indicating the presence of dentin. It’s noteworthy that the zone predominantly constituted by dentine reveals failures within the intended structure across several planes. This inference is supported by the micrographs presented in Fig. 7, where both longitudinal (Fig. 7b) and transverse (Fig. 7a) sections of dentinal tubules are observed.

**Fig. 8:**
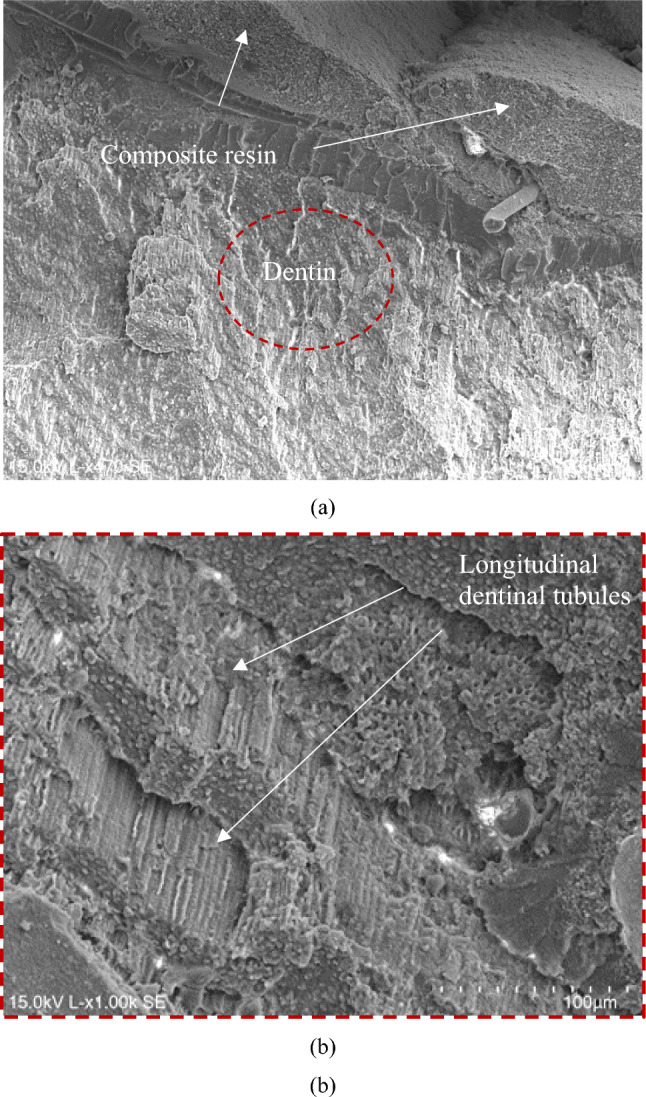
SEM micrographs of B3 specimen from the Papacárie Duo^®^ group after the shear bond strength test: **a** adhesive joint region (×470); **b** detail showing the dentinal tubules (×1.00 k)

As depicted in Fig. 8a, a similar phenomenon was observed for the specimens from the Papacárie Duo^®^ group. Areas where the composite resin predominates can be identified, along with other areas predominantly constituted by dentin, indicative of a mixed failure mode. Figure 8b, akin to the group comprising the Brix 3000™ specimens, fractures in the dentin are evident across several planes, revealing longitudinal and transverse sections of the dentinal tubules. Notably, this micrograph corresponds to the circular dashed area marked in Fig. 8a.

**Fig. 9:**
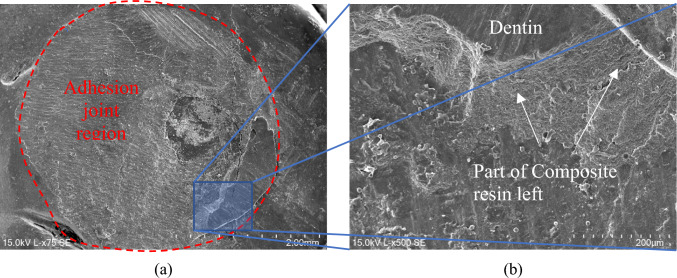
SEM micrographs showing the adhesive joint after the shear bond strength test for specimen C6 from the Control group: **a** 75 × magnification; **b** ×500 magnification

**Fig. 10:**
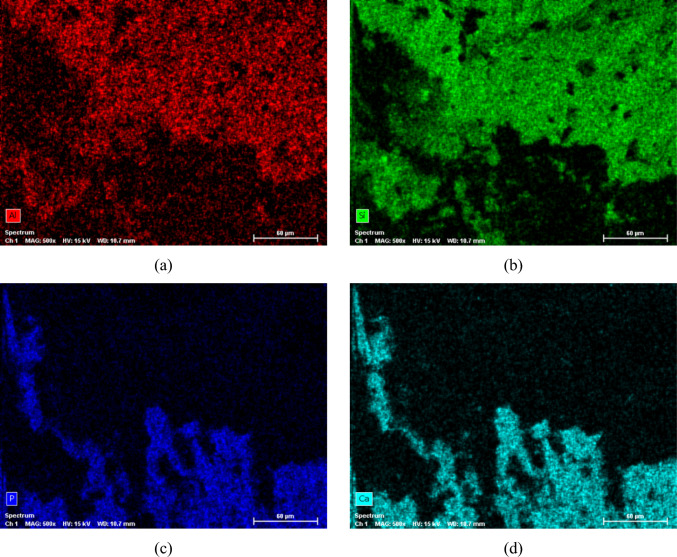
SEM/EDS mapping for the micrograph presented in Fig. 9b, showing the elements’ distribution at the adhesive joint for the Control group after the shear bond strength test (specimen C6): **a** Al distribution; **b** Si distribution; **c** P distribution; **d** Ca distribution

Figure 9a presents the bonded joint, marked by the circular area (red circle), following the shear bond strength test for sample C6 from the Control group. Figure 9b provides a magnified view of the area highlighted in Fig. 9a, showcasing a substantial amount of composite resin adhered to the dentin surface, indicative of a mixed failure. Figure 10 illustrates the EDS mapping, delineating the chemical elements present in the various regions.

All surfaces of the specimens analysed using SEM/EDS exhibited mixed failures, wherein it was possible to discern, within the fracture zone, an area comprised of dentin and another composed of composite resin. No cohesive or adhesive failures were observed, where only one of the two materials constituted the bonded joint is identifiable. The analysis exclusively targeted the dentin surface side.

## Discussion

According to the results of this in vitro study, the null hypothesis was partially rejected. Statistically significant differences were observed between the control group and the Brix 3000™ group regarding SBS, but not between the control group and the Papacárie Duo^®^ group. Regarding secondary outcomes, the Papacárie Duo^®^ group exhibited significantly higher work-to-debonding results, while the control group demonstrated significantly higher shear modulus results.

Several factors can influence shear bond strength results, including the substrate (enamel, deep affected dentin, superficial affected dentin), and the type of caries lesion (natural or artificially induced) (Nair et al. 2018).

The choice of substrate and the handling and storage of specimens for this experimental protocol followed ISO/TS 11405:2015 (Dental materials—testing of adhesion to tooth structure) guidelines (International Organization for Standardization 2015). The cavity needed to extend at least halfway from the enamel-dentin junction to the pulp chamber to minimize variability between specimens. The study’s teeth were stored in distilled water for a maximum period of six months to minimize substrate degeneration.

Artificially demineralized dentin exhibits collapsed collagen fibers and lacks vital odontoblasts capable of depositing minerals in the dentinal tubules, resulting in modified permeability. This modification can alter the hybridization of the dentin and the adhesive layer, impacting shear bond strength. Conversely, naturally decayed dentin presents greater variability in microorganisms, hydrolytic enzymes, and mineral deposition in the dentinal tubules as a response to the progression of the caries lesions. These characteristics make affected dentin less permeable, preventing the penetration of resin monomers, potentially affecting adhesion (Kusumasari et al. 2021; Nair et al. 2018).

When compared to carious dentin, healthy dentin exhibits structural histomorphological differences (such as a more stable and less humid collagen structure) and distinct mechanical properties (including greater hardness due to the superior mineral content) (Kusumasari et al. 2021; Nair et al. 2018). Although ISO/TS 11405:2015 (International Organization for Standardization 2015) recommends the use of healthy teeth in such studies, considering the specific objectives of the present study and the differing characteristics of dentin, carious teeth were selected to simulate the clinical substrate. This approach has been previously reported by other authors (Hamama et al. 2015; Kusumasari et al. 2021; Nair et al. 2018; Sajjad et al. 2022).

In addition to substrate differences, other variables such as the type of instrument used in the control groups for caries mechanical removal (such as excavator, disk, or bur), can influence the results. The use of different materials for carious tissue removal results in a remaining substrate with varying hardness and morphologically distinct smear layers, leading to differences in dentinal tubules permeability (Amaral et al. 2011). In the present study, a bur was used in the control group, as rotary instruments are the most commonly employed materials for caries removal (Corrêa et al. 2007; Hamama et al. 2015; Nair et al. 2018).

Regarding the method chosen to measure various adhesion parameters, it’s essential to critically analyse the available options. In scientific literature, two primary approaches are commonly discussed: the micro-tensile test (Pashley et al. 1999) and the shear bond strength test (Ilie et al. 2014; Alshali et al. 2021; Davalloo et al. 2020; Vivanco et al. 2020). The micro-tensile test offers the advantage of allowing a larger number of specimens for analysis. However, its main drawback is that the direction of load application (traction) does not align with the predominant forces experienced by restored teeth, primarily characterized by shear and compression. Consequently, the micro-tensile test tends to be more of a comparative method than one reflecting real-world scenarios. On the other hand, shear tests closely mimic real-world scenarios, providing more realistic shear strength values. Nonetheless, each test requires a single tooth, which can pose challenges in specimen collection. In the present study, the authors opted for the shear bond strength test, a decision well-suported from a scientific perspective.

Several studies have investigated the chemical and morphological changes occurring on residual dentin surfaces following enzymatic chemo-mechanical caries removal. It has been reported that the application of Papacárie^®^/Papacárie Duo^®^ on dentin leads to surface demineralization, resulting in the formation of irregularities and porosities. Authors have observed that this treatment partially or completely opens dentinal tubules, contrasting with outcomes observed with rotary instruments (Corrêa et al. 2007; Kusumasari et al. 2021; Sajjad et al. 2022). Furthermore, the deproteinization of the smear layer induced by Papacárie^®^/Papacárie Duo^®^ facilitates the removal of damaged collagen fibers, leaving behind stable collagen that facilitates optimal chemical bonding with dental adhesives (Kusumasari et al. 2021). Microscopic analysis has revealed either a minimal or absent smear layer or a thinner and more uniform amorphous layer post Papacárie^®^/Papacárie Duo^®^ application, in comparison to the layer produced by rotatory instruments (Corrêa et al. 2007; Hamama et al. 2013; Kusumasari et al. 2021).

These unique characteristics contribute to an increased adhesive area, potentially enhancing micromechanical retention and promoting more effective penetration of resin monomers (Hamama et al. 2013; Nair et al. 2018). Consequently, it was anticipated that Papacárie Duo^®^ would yield higher shear bond strength compared to conventional caries removal methods. However, while the present study did not demonstrate significantly higher shear bond strength, the absence of statistically significant differences between the Papacárie Duo^®^ group and the control group aligns with findings reported by other researchers (Amaral et al. 2011; Hamama et al. 2014, 2015; Lopes et al. 2006; Nair et al. 2018).

Although Brix 3000™ also contains papain in its formulation, Donmez et al. (2022) described the development of a thin hybrid layer, devoid of a smear layer, after its application on dentin. This contrasts with the outcomes associated with Papacárie Duo^®^, suggesting potential differences in the proteolytic activity between the two products. The higher concentration of papain in Brix 3000™ may contribute to its stronger proteolytic activity.

The high proteolytic activity of papain, acting on denatured collagen molecules, may facilitate the removal of infected dentin. The presence of the antiprotease alpha-1-antitrypsin in healthy tissues serves a protection role by inhibiting the proteolytic activity of the material. Therefore, only the tissue with denatured collagen fibers (infected dentin) is targeted for removal, while the affected dentin capable of regeneration remains preserved (Santos et al. 2020). Given these mechanisms, the inferior results obtained after using Brix 3000™ may be attributed not only to its higher papain concentrations but also to its extended application time on dentin (2 min compared to 30 s for Papacarie Duo^®^), potentially intensifying its proteolytic activity. Further studies, particularly cellular investigations, are needed to comprehensively assess its effect on the dentin substrate.

However, the results of the present in vitro study indicate that Brix 3000™ significantly compromises adhesion to dentin. No other studies evaluating adhesion to dentin after Brix 3000™ application were identified.

Regarding work-to-debonding, it was noted that the Papacárie Duo^®^ group exhibited higher values than the other two groups. Therefore, it can be inferred that Papacárie Duo^®^ induced structural changes in dentin, enhancing its capacity to absorb energy when subjected to repetitive stresses, thereby minimizing debonding or detachment.

The control group, characterized by a higher stiffness in the adhesive system – composite resin joint, exhibits fracture/detachment at lower displacement values compared to the other groups. Despite the Papacárie Duo^®^ group demonstrating inferior rigidity, it displays higher work-to-debonding than the Brix 3000™ group, suggesting that Papacárie Duo^®^ induces structural changes in the tooth, thereby enhancing displacement and energy absorption without rupture.

In summary, while the control group necessitates greater force for rupture, the Papacárie Duo^®^ group manifests greater tenacity, absorbing a larger amount of energy represented by the area under the stress–strain curve without experiencing rupture. This indicates that the control group exhibits a more brittle behaviour, whereas the Papacárie Duo^®^ group demonstrates a more tenacious behaviour.

Like many other studies, this research also encounters limitations that warrant consideration. The primary constraint lies in its study type, which is confined to an in vitro setting. Consequently, the generalization and extrapolation of results to clinical contexts are restricted. Another limitation pertains to the relatively modest sample size (*n* = 30) and the absence of sample size calculation or power analysis. Additionally, there may be concerns regarding standardization when employing natural caries lesions. To address these limitations, further research with larger sample sizes and robust methodologies are warranted to validate and extend this study’s findings. Specifically, exploring the efficacy and safety of Brix 3000™ and Papacárie Duo^®^ through in vitro, cellular, and clinical studies would be beneficial. Additionally, investigating the performance of these products on different dentin substrates (superficial, deep, healthy, and sclerotic dentin, for example), and with various adhesive systems (self-etch, total-etch, and universal), and composite resin systems (nano/micro hybrid/particle) would provide a more comprehensive understanding of their effectiveness and suitability for clinical use.

## Conclusion

Considering any limitations of the present study, it has been shown that enzymatic chemo-mechanical caries removal using Papacárie Duo^®^ did not compromise the adhesion of dentin of permanent teeth to composite resin in vitro. Papacárie Duo^®^ also exhibited similar shear bond strength to the control group and higher work-to-debonding values among the three groups.

The use of Brix 3000™ enzymatic chemo-mechanical caries removal agent resulted in lower shear bond strength and shear modulus of composite resin to dentin of permanent teeth, suggesting a potential limitation of this method for caries removal.

Further investigations are warranted to elucidate the effects of these materials on dentin substrate, particularly through cellular and clinical studies. Despite limitations inherent to in vitro research, such as restricted generalizability, this study underscores the need for comprehensive evaluations of adhesive systems and caries removal methods to enhance clinical outcomes.

## Author contributuions

All authors contributed to the study conception and design. Material preparation, data collection and analysis were performed by Ana Coelho, Luís Vilhena, Inês Amaro, Ana Melo, Amílcar Ramalho and Eunice Carrilho. The first draft of the manuscript was written by Ana Coelho, Luís Vilhena, Inês Amaro, Ana Melo, Anabela Paula, Carlos Miguel Marto and Manuel Marques Ferreira. All authors commented on previous versions of the manuscript. All authors read and approved the final manuscript.
